# Involvement of Mechanistic Target of Rapamycin (mTOR) in Valine Orexigenic Effects in Rainbow Trout

**DOI:** 10.1155/2022/7509382

**Published:** 2022-09-27

**Authors:** Sara Comesaña, Mauro Chivite, Ayelén M. Blanco, María Alborja-Valado, Jessica Calo, Marta Conde-Sieira, José L. Soengas

**Affiliations:** Centro de Investigación Mariña, Laboratorio de Fisioloxía Animal, Departamento de Bioloxía Funcional e Ciencias da Saúde, Facultade de Bioloxía, Universidade de Vigo, E-36310 Vigo, Spain

## Abstract

This study was aimed at clarifying the importance of a mechanistic target of rapamycin (mTOR) in the central orexigenic effect of valine in fish. For this, rainbow trout (*Oncorhynchus mykiss*) were intracerebroventricularly (ICV) injected with valine alone or in the presence of rapamycin as the mTOR inhibitor, and two experiments were performed. In the first experiment, we evaluated feed intake levels. In the second experiment, we evaluated in the hypothalamus and telencephalon the following: (1) the phosphorylation status of mTOR and its downstream effectors ribosomal protein S6 and p70 S6 kinase 1 (S6K1), (2) the abundance and phosphorylation status of transcription factors involved in appetite regulation, and (3) the mRNA levels of key neuropeptides associated with homeostatic regulation of feed intake in fish. Rising central levels of valine clearly resulted in an orexigenic response in rainbow trout. This response occurred in parallel with mTOR activation in both the hypothalamus and telencephalon, as supported by depressant changes in proteins involved in mTOR signalling (S6 and S6K1). Also, these changes disappeared in the presence of rapamycin. However, it is not clear which precise mechanisms link the activation of mTOR and the alteration in feed intake levels since we did not observe changes in mRNA levels of appetite-regulatory neuropeptides as well as in the phosphorylation status and levels of integrative proteins.

## 1. Introduction

Mechanistic target of rapamycin (mTOR) is a protein kinase integrating diverse signals to control cell growth potentiating anabolic processes [1, 2]. In the hypothalamus, mTOR receives signals from nutrients and hormones to regulate energy homeostasis through energy expenditure and feed intake [3]. In this brain area, two populations of neurons are sensitive to those regulatory signals and consequently regulate expression of several neuropeptides in order to modulate feed intake: first, the orexigens agouti-related peptide (AgRP) and neuropeptide Y (NPY) that increase feed intake; second, amphetamine- and cocaine-related transcript (CART) and proopiomelanocortin (POMC) that decrease feed intake [4]. The expression of these neuropeptides is mediated by the activity of mTOR. Thus, nutrients, and especially amino acids, induce the activation of mTOR in order to decrease AgRP and NPY levels, while increasing those of CART and POMC finally resulting in lower feed intake [3].

In mammals, the branched-chain amino acid (BCAA) leucine has been reported to stimulate the mTOR signalling pathway [5]. Additionally, leucine is the unique BCAA related to food intake regulation [6]. Thus, mTOR and leucine are apparently linked in the context of food intake regulation [3]. Actually, suppression of mTOR prevents leucine from reducing food intake [7, 8].

In fish, mTOR was characterized in the hypothalamus (and other brain areas) of species like rainbow trout [9–11] and Japanese sea bass (*Lateolabrax japonicus*) [12]. In rainbow trout, levels and phosphorylation status of mTOR increase in response to the presence of nutrients such as glucose [11], oleate [9, 11], or octanoate [9]. In addition, the mTOR inhibitor rapamycin has been observed to block the anorexigenic effects that fatty acids elicit in the rainbow trout hypothalamus [9], supporting the specificity of the response. Leucine also elicits an anorexigenic effect in fish, as demonstrated in rainbow trout [10], Atlantic salmon (*Salmo salar*) [13], tiger puffer (*Takifugu rubripes*) [14], Chinese perch (*Siniperca chuatsi*) [15], zebrafish (*Danio rerio*) [16], or hybrid catfish (*Pelteobagrus vacheli* × *Leiocassis longirostris*) [17]. These responses in feed intake occur in parallel with increased mTOR in the hypothalamus and telencephalon, as demonstrated in rainbow trout [10]. In contrast to the mammalian model, there is evidence in fish for another BCAA, namely, valine, eliciting changes in feed intake. Surprisingly, these changes reflect an orexigenic rather than an anorexigenic response, as characterized in rainbow trout [10] and Chinese perch [15]. However, the mechanisms underlying the appetite-stimulatory actions of valine in fish remain to be elucidated.

In this study, we aimed to clarify the role of mTOR in the stimulation of feed intake observed after central administration of valine in rainbow trout (*Oncorhynchus mykiss*). For this, we carried out an intracerebroventricular (ICV) injection of valine alone or in the presence of the mTOR inhibitor rapamycin. In the first experiment, we evaluated feed intake levels. Then, in the second experiment, we carried out the same treatment and evaluated in the hypothalamus and telencephalon the following: (1) the phosphorylation status of mTOR and its downstream effectors ribosomal protein S6 and p70 S6 kinase 1 (S6K1); (2) the protein abundance and the status of phosphorylation of the transcription factors related to feed intake regulation [18] such as brain homeobox transcription factor (Bsx), forkhead box01 (Foxo1), and cAMP response-element binding protein (Creb); and (3) the abundance of mRNAs encoding the main neuropeptides associated with homeostatic regulation of feed intake in fish, namely, *agrp*, *npy*, *cartpt*, and *pomc* [19].

## 2. Materials and Methods

The study complied with the regulations of the Spanish Government (RD53/2013) and European Union Council (UE63/2010) for animal use in scientific research following guidelines of the Declaration of Helsinki. Universidade de Vigo Ethics Committee approved the procedures (CEEA-O.H.-UVI-00013-19JLSF), which were authorized by Xunta de Galicia Regional Government (ES360570181401/19/FUN01/FIS02/JLSF01). Some of the methods mentioned below have been previously described in the PhD thesis of the first author Sara Comesaña [20].

### 2.1. Fish

Rainbow trout (37.4 ± 1.1 g body weight) were obtained in Piscifactoría de la Calle (fish farm located in A Estrada, Spain) and maintained 1 month in the facilities of Universidade de Vigo. They were kept in 100-litre tanks in tap water (dechlorinated) at 15°C of temperature and under a photoperiod of 12 h light : 12 h darkness. Fish were fed to satiety once daily using commercial pelleted diet from Biomar (Dueñas, Spain) containing 44% crude protein, 17% ash, 2.5% carbohydrates, and 21% crude fat.

### 2.2. Experimental Design

After one month of acclimation, fish were (randomly) distributed into 100-litre experimental tanks and were deprived of feed for 24 h before treatment. The day of the experiment, fish were anaesthetized (2-phenoxyethanol; Sigma, 0.02% *v*/*v*) and weighed. Then, we carried out ICV administration, following a previously described method [21]. We dispensed 1 *μ*L·100 g^−1^ body mass of saline solution and dimethyl sulfoxide (DMSO) alone (control) or containing 20 mM of rapamycin (R), 80 mM of L-valine (V), or rapamycin with valine (R+V). The dose of rapamycin was selected based on studies performed in mammals [7, 22] and validated in rainbow trout [9, 23]. We selected the dose of valine based on prior studies with rainbow trout [10].

In the first experiment, we assessed the basal level of feed intake for 3 days before treatment. Then, we assessed feed intake 6, 24, and 48 h after ICV treatment with saline solution and DMSO alone (control) or containing L-valine, rapamycin, or both. After feeding, the uneaten feed was recovered from the tank bottom, dried, and weighed. This value was used to calculate feed consumed by all fish in each tank [24]. The experiment was repeated three times. The results are shown as the mean ± SEM of 3 different experiments (*N* = 3) with 12 fish per treatment (*n* = 12).

In the second experiment, fish were ICV injected as described above with saline solution and DMSO alone (control, *n* = 12) or containing rapamycin (*n* = 12), L-valine (*n* = 12), or both (*n* = 12). After 6 h, fish were lightly anaesthetized with 0.02% *v*/*v* 2-phenoxyethanol. Then, fish were sacrificed (decapitation), and subsequently, brain regions (hypothalamus and telencephalon) were dissected to be snap-frozen and stored for further analysis at -80°C. We used six fish per group to assess changes in mRNA abundance by qRT-PCR. We used the remaining six fish per group to evaluate levels of specific proteins by Western blot.

### 2.3. Analysis of mRNA Abundance by RT-qPCR

We extracted total RNA with TRIzol (Life Technologies, Grand Island, NY, USA), and then, samples were treated with RQ1-DNAse (Promega, Madison, WI, USA) following the manufacturer's instructions. Total RNA (2 *μ*g) was reverse-transcribed in a 25 *μ*L reaction volume with random primers (Promega) and M-MLV reverse transcriptase (Promega). The levels of mRNA of key neuropeptides were assessed by RT-qPCR using the CFX Connect Real-Time PCR Detection System (Bio-Rad, Hercules, CA, USA). The analysis was performed in a reaction volume of 15 *μ*L containing 2 *μ*L cDNA from each sample (1 : 4 previous dilution), 0.5 *μ*M of primers (forward and reverse, Table 1), and Maxima SYBR Green qPCR Master Mix (Life Technologies). The thermal cycling consisted of (1) hot start iTaq DNA polymerase incubation for 10 min at 95°C, (2) 40 heating cycles of 15 s each at 95°C for 15 s, and (3) 40 s of annealing and extension at 60°C. When finished, we performed melting curves (0.5°C/5 s temperature gradient from 65 to 95°C) to guarantee the amplification of one fragment. We also carried out two types of negative controls in each qPCR, i.e., samples without RNA and samples without RT. We calculated the relative mRNA abundance of evaluated transcripts using *actb* (*β*-actin) and *eef1a1* (elongation factor 1*α*) as reference transcripts [25].

### 2.4. Western Blot Analysis

Frozen samples were homogenized in 0.5 mL of buffer formed by 100 mM NaF, 150 mM NaCl, 2 mM Na_3_VO_4_, 4 mM Na_4_P_2_O_7_, 1% Triton X-100, 1 mM EGTA, 1 mM EDTA, 1.02 mg/mL protease inhibitor cocktail (Sigma), 0.5% NP40-IGEPAL, and 10 mM Tris-HCl (pH 7.4). We first centrifuge homogenates (1000 × g for 15 min at 4°C). Then, supernatants were centrifuged again (20,000 × g for 30 min at 4°C), recovered, and stored (−80°C) for subsequent analysis. We evaluated protein concentration in each sample following the Bradford method (using as standard bovine serum albumin from Sigma). Then, we performed western blots with 20 *μ*g of protein lysates and different primary antibodies (Table 2), previously validated in rainbow trout [26–31]. After washing, we incubated membranes with a IgG-HRP secondary antibody from Abcam (ref. #2015718 with a 1 : 5000 dilution). Band densities were calculated (two gels per protein of interest and tissue) in a ChemiDoc Touch Imaging system with Image Lab software version 5.2.1 (Bio-Rad). Original blots are included as supplementary Figure 1.

### 2.5. Statistics

Data are expressed as mean ± SEM. We checked data for variance homogeneity and normality. If required, data were log-transformed and rechecked. Groups were compared with one-way ANOVA followed by a Bonferroni test using the statistical package SigmaStat (Systat Software Inc., San Jose, California, USA). Differences were considered significant at *P* < 0.05.

## 3. Results

**Figure 1:**
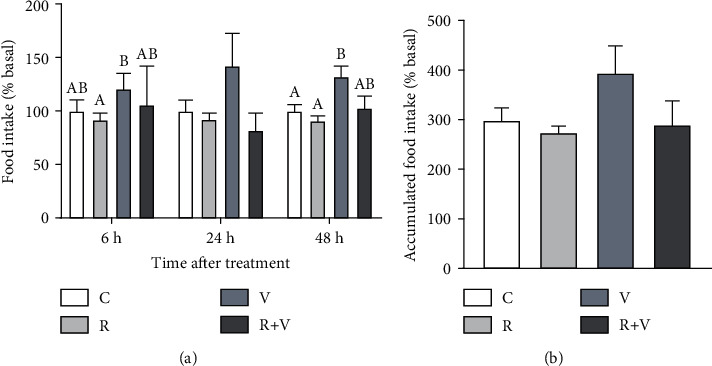
Feed intake was measured in rainbow trout after 6, 24, and 48 h of intracerebroventricular injection of 1 *μ*L·100 g^−1^ body mass of a solution containing saline and dimethyl sulfoxide (DMSO) alone (control) or containing 20 mM rapamycin (R), 80 mM of L-valine (V), or rapamycin with valine (R+V). (a) Feed intake is shown as a percentage of ingested feed compared with basal levels (average of intake 3 days prior to experiment) and normalized to the control group (100%). Values are mean + SEM of feed intake assessed in 3 different tanks (12 fish per tank) per treatment. Significant differences (*P* < 0.05) among groups are denoted with different letters. (b) Accumulated feed intake is shown as mean + SEM of the summatory of the percentage of feed ingested calculated each time.

In the first experiment, feed intake showed a general increase after valine treatment, being significantly different in comparison to rapamycin group at 6 h postinjection and different to control at 48 h (Figure 1(a)). A similar tendency of feed intake levels being higher upon valine treatment is observed when accumulated feed intake is represented, although there are no statistically significant differences (Figure 1(b)). Rapamycin alone or with valine did not affect feed intake.

**Figure 2:**
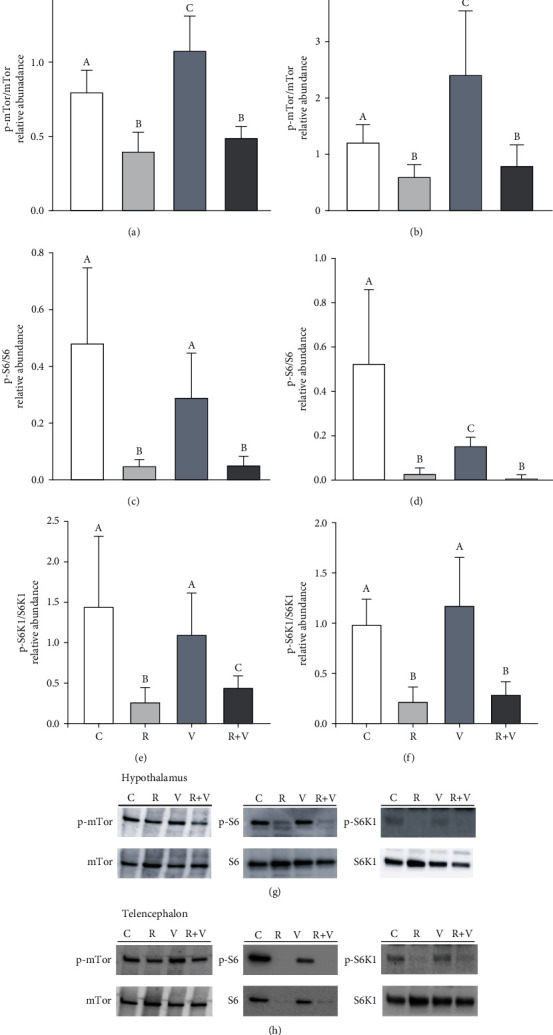
Phosphorylation status of mTOR (a, b), S6 (c, d), and S6K1 (e, f) in the hypothalamus (a, c, e) and telencephalon (b, d, f) of rainbow trout 6 h after intracerebroventricular injection of 1 *μ*L·100 g^−1^ body mass of a solution containing saline and dimethyl sulfoxide (DMSO) alone (control) or containing 20 mM rapamycin (R), 80 mM of L-valine (V), or rapamycin with valine (R+V). Values are mean + SEM of *N* = 6 fish per treatment. One representative blot per treatment is shown for the hypothalamus (g) and telencephalon (h). Significant differences (*P* < 0.05) among groups are denoted with different letters.

In the second experiment, both in the hypothalamus and telencephalon, valine treatment increased the phosphorylation of mTOR, while a decrease was observed upon treatment with rapamycin alone or in combination with valine (Figures 2(a)and 2(b)). Phosphorylation status of S6 decreased with rapamycin and rapamycin+valine treatments in the hypothalamus (Figure 2(c)) and telencephalon (Figure 2(d)). The treatment with valine alone did not affect S6 phosphorylation in the hypothalamus (Figure 2(c)), while its administration in the telencephalon led to lower values of p-S6/S6 levels compared with controls but higher than the other groups (Figure 2(d)). Decreased phosphorylation status of S6K1 occurred in the hypothalamus and telencephalon after treatment with rapamycin and rapamycin+valine, while valine treatment alone did not elicit any change in comparison to the control group (Figures 2(e) and 2(f)).

**Figure 3:**
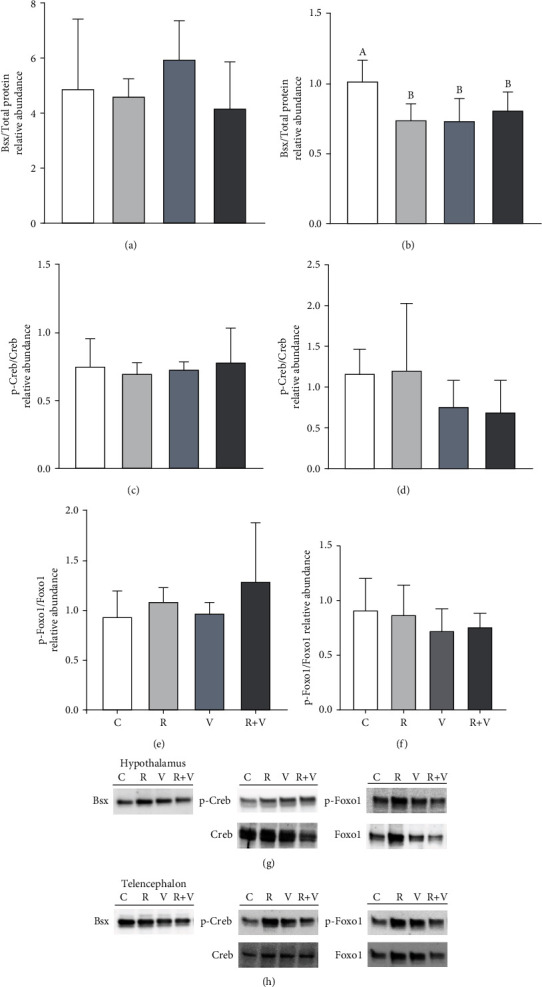
Abundance of Bsx (a, b) and phosphorylation status of Creb (c, d) and Foxo1 (e, f) in hypothalamus (a, c, e) and telencephalon (b, d, f) of rainbow trout 6 h after intracerebroventricular injection of 1 *μ*L·100 g^−1^ body mass of a solution containing saline and dimethyl sulfoxide (DMSO) alone (control) or containing 20 mM rapamycin (R), 80 mM of L-valine (V), or rapamycin with valine (R+V). Values are mean + SEM of *N* = 6 fish per treatment. One representative blot per treatment is shown for the hypothalamus (g) and telencephalon (h). Significant differences (*P* < 0.05) among groups are denoted with different letters.

Figure 3 shows the protein abundance of Bsx (Figures 3(a) and 3(b)) and the phosphorylation status of Creb (Figures 3(c) and 3(d)) and Foxo1 (Figures 3(e) and 3(f)) in response to the ICV administration of valine and rapamycin. We only observed significant differences in the relative abundance of Bsx in telencephalon, where all treatments reduced Bsx protein levels compared to the control group (Figure 3(d)).

**Figure 4:**
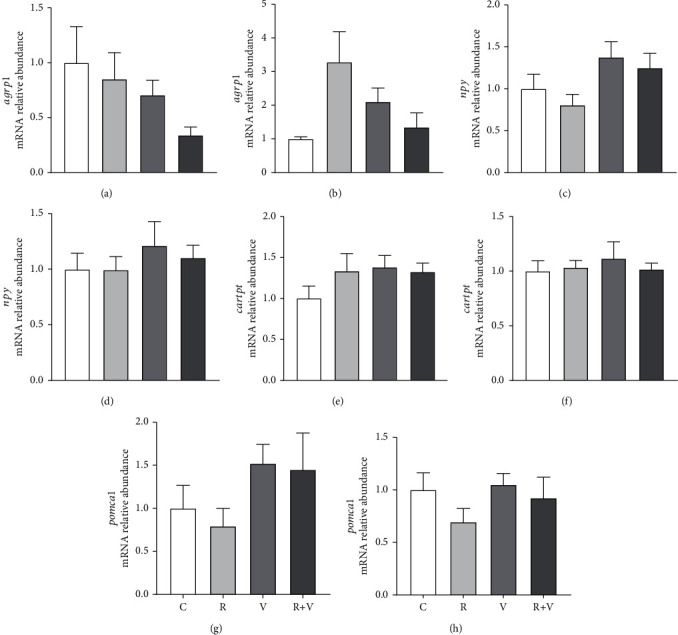
mRNA relative abundance of the *agrp1* (a, b), *npy* (c, d), *cartpt* (e, f), and *pomca1* (g, h) in the hypothalamus (a, c, e, g) and telencephalon (b, d, f, h) of rainbow trout 6 h after intracerebroventricular injection of 1 *μ*L·100 g^−1^ body mass of a solution containing saline and dimethyl sulfoxide (DMSO) alone (control) or containing 20 mM rapamycin (R), 80 mM of L-valine (V), or rapamycin with valine (R+V). Values (referred to as the control group) are mean ± SEM of *N* = 6 fish per treatment and were previously normalized by mRNA abundance of *actb* and *eef1a1* mRNA.

Finally, the treatments did not induce significant changes in mRNA abundance of neuropeptides (Figure 4).

## 4. Discussion

In the present study, we observed an orexigenic effect upon ICV treatment with valine in rainbow trout. This result agrees with a similar previous finding observed in rainbow trout [10], thus supporting the experimental design used, as well as with a result obtained in another fish species, the Chinese perch [15]. These observations are quite surprising from the mammalian point of view, where valine treatment does not affect feed intake in comparison with leucine [3, 32]. In our previous study observing an appetite-stimulatory role for valine, we also found evidence for the existence in rainbow trout hypothalamus and telencephalon of different amino acid-sensing systems, which clearly responded to the increase in the levels of leucine, finally resulting in an anorexigenic response to amino acid [10]. However, we observed no changes in the same systems in response to valine [10] allowing us to suggest that the orexigenic response of this amino acid might relate to factors other than direct amino acid sensing.

Therefore, we here evaluated the putative involvement of mTOR in the orexigenic response of rainbow trout to valine by centrally treating fish with this amino acid alone or in the presence of the mTOR inhibitor rapamycin, known to inhibit the anorexigenic effects of leucine in mammals [3]. In fish, this inhibitor has been previously used to block mTOR response in rainbow trout [9], Chinese perch [15], goldfish (*Carassius auratus*) [33], or zebrafish [34]. Results from our study pointed out that rapamycin itself has no impact on feed intake but clearly inhibits levels and phosphorylation status not only of mTOR but also of its downstream proteins such as S6 and S6K1 both in the hypothalamus and telencephalon. This strongly demonstrates that the inhibitor is altering the mTOR pathway, thus again supporting the validity of the experimental design.

Treatment with valine raised in the hypothalamus and telencephalon the phosphorylation status of mTOR, suggesting its involvement in the response to the presence of valine in central areas of fish. This is the first time in which such a response has been observed in specific brain regions of any fish species since available evidence regarding ICV valine treatment dealt with changes in the mTOR system either in the whole brain [15] or in liver [35] of Chinese perch. Interestingly, the effect of centrally administered valine on the mTOR system in the liver of Chinese perch was an activation comparable to that herein observed in brain areas of rainbow trout. A previous study in hybrid grouper (*Epinephelus fuscoguttatus* × *Epinephelus lanceolatus*) also reported that increased valine elicits activation of mTOR in the liver [36]. Our results point towards the increase in mTOR phosphorylation upon valine treatment being specific since it was not observed when fish were simultaneously treated with the inhibitor rapamycin. In addition, we observed that the presence of rapamycin clearly blocked the effect of valine on the proteins involved downstream in the response of mTOR, resulting in values considerably lower than those of the valine treatment alone. Altogether, changes observed in this study clearly support the involvement of mTOR in the physiological actions of valine in rainbow trout.

However, the activation of mTOR response typically occurs under anorexigenic and not orexigenic conditions, as demonstrated in different situations both in mammals [37, 38] and in fish [9–11, 31]. Indeed, a comparable activation of mTOR was observed in rainbow trout in response to ICV treatment with the appetite-inhibitory amino acid leucine [10]. We therefore aimed to clarify these apparent discrepancies and to obtain more information regarding the intracellular mechanisms underlying the orexigenic effect of valine in trout. Thus, we also evaluated changes in the levels and phosphorylation status of several of the proteins involved in the integration of metabolic and endocrine signalling in brain resulting in changes in feed intake such as those of Bsx, Creb, and FoxO1 [18]. Except for a minor response in Bsx levels in telencephalon, we failed to observe major changes in these proteins (both levels and phosphorylation status). This is in contrast to changes previously observed in rainbow trout in response to leucine treatment, which altered these proteins in a way indicative of a clear anorexigenic response [18]. The response to valine is comparable to that previously observed in rainbow trout [10] but what is also evident in the present study is the lack of effect of rapamycin. The lack of valine-induced changes in integrative proteins in the hypothalamus and telencephalon allows us to suggest the involvement of alternative pathways being involved in the orexigenic effect of valine.

As a final step in the regulatory pathways governing feed intake, we evaluated the impact of valine treatment on the abundance of mRNAs encoding key neuropeptides involved in the final integratory response to the presence of nutrients and hormones in the brain, namely, *agrp1, npy, pomca1*, and *cartpt*, as repeatedly demonstrated in different fish species including rainbow trout [18, 19]. As with the protein levels above, no major changes were observed in the relative mRNA levels of these neuropeptides upon valine treatment in this study. Again, this is similar for the effect of valine treatment alone [10] but novel for the absence of effects of rapamycin. The absence of valine effects in neuropeptide mRNA abundance, together with the absence of changes in integrative proteins, seems to indicate that the main integrative responses typically occurring during the homeostatic regulation of feed intake are not taking place in the rainbow trout's hypothalamus and telencephalon when valine was present. However, we should not forget that only mRNA abundance was evaluated in the case of neuropeptides and that mRNA levels are not always indicative of protein levels. While similar care should be taken for the absence of neuropeptide protein determination, the fact that rapamycin was not able to induce any changes in neuropeptide mRNA abundance allows us to suggest that these neuropeptides are not involved in the signalling directed by mTOR in response to valine. This is in clear contrast with the response of rainbow trout brain to leucine [10] where leucine-evoked changes in the mRNA abundance of neuropeptides occurred in a way similar to that described in mammals; i.e., the decrease in feed intake induced by leucine is mediated by changes in the mRNA abundance of CART, NPY, POMC, and AgRP [3]. This absence of changes in the relative abundance of neuropeptides mRNA is also different than that observed in response to raised levels of other nutrients like fatty acids whose presence activated not only mTOR but also changes in all parameters related to the homeostatic regulation of feed intake in fish, and the activation disappeared in the presence of rapamycin [9]. This lack of changes in integrative proteins and neuropeptide mRNA abundance is even more surprising considering that the activation we clearly observed in the mTOR response is the typical response involved in other situations in rainbow trout when a final decrease in feed intake also occurs, including increased levels of nutrients like fatty acid [9] or glucose [11]. This response is also similar to that observed under conditions eliciting anorectic responses in different fish species. These include feeding a lipid-enriched diet in rainbow trout [39] and blunt snout bream (*Megalobrama amblycephala*) [40] or different treatments in rainbow trout such as those with insulin [11], PYY [41], CCK [42], or ceramide [43]. In other fish species, increased mTOR activation occurred in parallel with anorexigenic and not orexigenic responses as observed in the hypothalamus of Japanese sea bass [12], the whole body of zebrafish [16], or in the muscle of hybrid catfish [17].

Clearly, mTOR response to valine is interacting with another pathway of feed intake regulation considering that a similar response to a rise in valine occurred for anorexigenic amino acids such as leucine, but the final response of the fish in terms of feeding is the converse. As suggested in a previous study [31], this might relate to hypothetical reward response elicited by valine in a way that this amino acid would relate to the hedonic rather than the homeostatic regulation of feed intake [19]. Nevertheless, this explanation is not so easy since in a previous study, we observed that this amino acid was not as attractive for this species compared with others [31]. If related to hedonic regulation of feed intake, the orexigenic effect of valine and the simultaneous activation of mTOR must relate to changes in different systems describing changes under reward conditions such as endocannabinoids and opioids [19]. The finding that valine treatment resulted in an anorexigenic effect when administered peripherally in the same species [31] is again suggesting some sort of unknown interaction after central valine treatment. Additional studies are necessary to fully elucidate the role of valine in feed intake regulation in fish.

## 5. Conclusions

In this study, we observed that rising central levels of valine clearly resulted in an orexigenic response in rainbow trout. This response in feed intake occurred at the same time that mTOR activation in both hypothalamus and telencephalon as also supported by changes in downstream proteins involved in mTOR signalling (S6 and S6K1) and the finding that changes disappeared in the presence of the mTOR antagonist rapamycin. However, it is not clear what precise mechanisms link the activation of mTOR and feed intake response since no changes occurred in (1) the relative mRNA levels of key neuropeptides associated with homeostatic regulation of feed intake [19] and (2) the levels and phosphorylation status of other integrative proteins [18]. Therefore, other alternative systems putatively related to the hedonic regulation of feed intake [19] must be involved in the effect of valine. Finally, the involvement of mTOR in an orexigenic response to valine is fish-specific since in mammals, this BCAA not only is inducing no changes in feed intake but is also considered as adverse compared with leucine [44]. Certainly, the situation in fish appears to be different, and this might relate to the high requirements of valine as an essential amino acid in fish, including rainbow trout [45].

## Figures and Tables

**Table 1:** Primer sequences used to evaluate mRNA abundance by quantitative RT-PCR (qPCR).

Transcript	Forward primer	Reverse primer	GenBank ID
*actb*	GATGGGCCAGAAAGACAGCTA	TCGTCCCAGTTGGTGACGAT	NM_001124235.1
*agrp1*	ACCAGCAGTCCTGTCTGGGTAA	AGTAGCAGATGGAGCCGAACA	CR376289
*cartpt*	ACCATGGAGAGCTCCAG	GCGCACTGCTCTCCAA	NM_001124627
*eef1a1*	TCCTCTTGGTCGTTTCGCTG	ACCCGAGGGACATCCTGTG	AF498320
*pomca1*	CTCGCTGTCAAGACCTCAACTCT	GAGTTGGGTTGGAGATGGACCTC	TC86162 (Tigr)
*npy*	CTCGTCTGGACCTTTATATGC	GTTCATCATATCTGGACTGTG	NM_001124266

*actb*: *β*-actin; *agrp1*: agouti-related peptide 1; *cartpt*: cocaine- and amphetamine-related transcript; *eef1a1*: elongation factor 1*α*; *pomca1*: proopiomelanocortin a1; *npy*: neuropeptide Y.

**Table 2:** Primary antibodies used to evaluate protein abundance by Western blot.

Company	Name	Reference	Dilution used
Cell Signaling Technology (Leiden, Netherlands)	Anti-CREB (48H2)	#9197	1 : 500
	Anti-phospho-CREB (Ser133)	#9198	1 : 500
	Anti-FoxO1 (L27)	#9454	1 : 500
	Anti-phospho-FoxO1 (Thr24)	#9464	1 : 500
	Anti-S6	#2217	1 : 500
	Anti-phospho-S6 (Ser235/236)	#4856	1 : 500
	Anti-S6K1	# 9202	1 : 500
	Anti-phospho-S6K1	#9205	1 : 500
	Anti-phospho-mTOR (Ser2448)	#5536	1 : 500
	Anti-*β*-tubulin	#2146	1 : 2000

Sigma-Aldrich (St. Louis, USA)	Anti-mTOR	#T2949	1 : 500

Abcam (Cambridge, UK)	Anti-BSX	#ab56092	1 : 250

## Data Availability

The data used to support the findings of this study are available from the corresponding author upon request.

## Supplementary Materials

Supplementary Materials Original blots are included as supplementary Figure 1.
