# Supplementary material for: Involvement of Mechanistic Target of Rapamycin (mTOR) in Valine Orexigenic Effects in Rainbow Trout
Source: Aquac Nutr. 2022 Sep 27;2022:7509382. doi: 10.1155/2022/7509382 (PMC9973124; doi:10.1155/2022/7509382)

Supplementary Figure 1. Original gels used in Western blots

Two gels were used per potein in each tissue. Each of them contained 3 samples from each group: control (C), rapamycin (R), valine (V) and rapamyicin+Valine (RV). The sum of the two gels result in 6 samples per group as described in Materials and Methods

Bsx Hypothalamus (36 kDa)

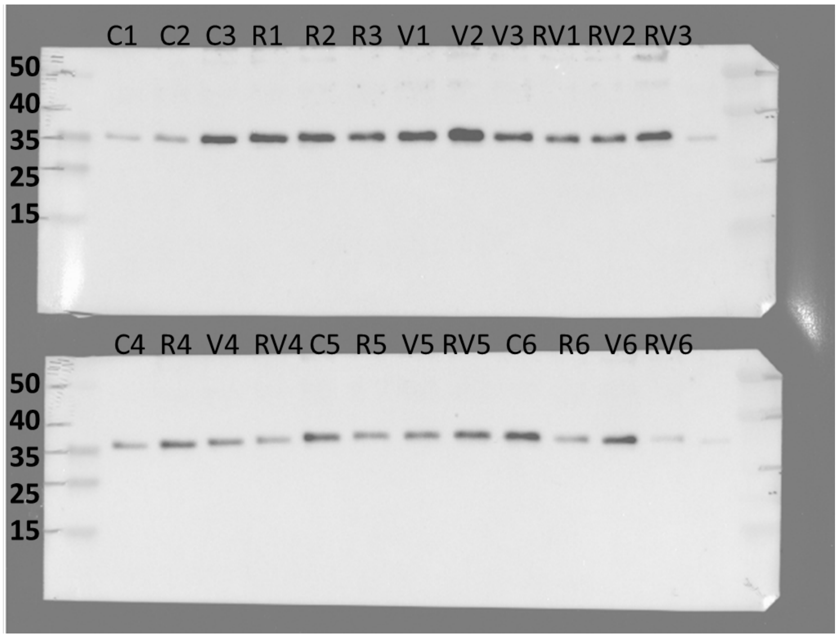

Bsx Telencephalon (36 kDa)

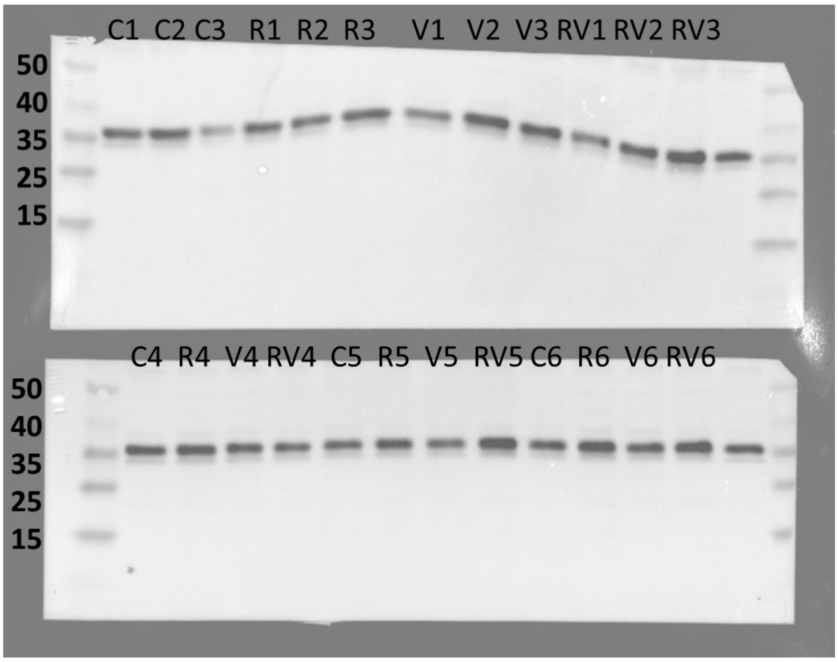

p-Creb Hypothalamus (43 kDa)

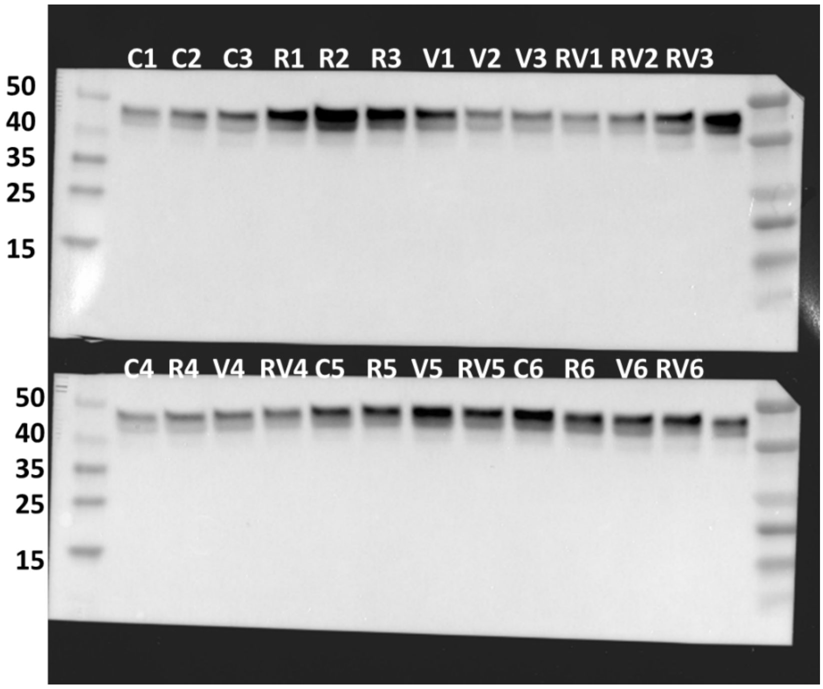

Creb Hypothalamus (43 kDa)

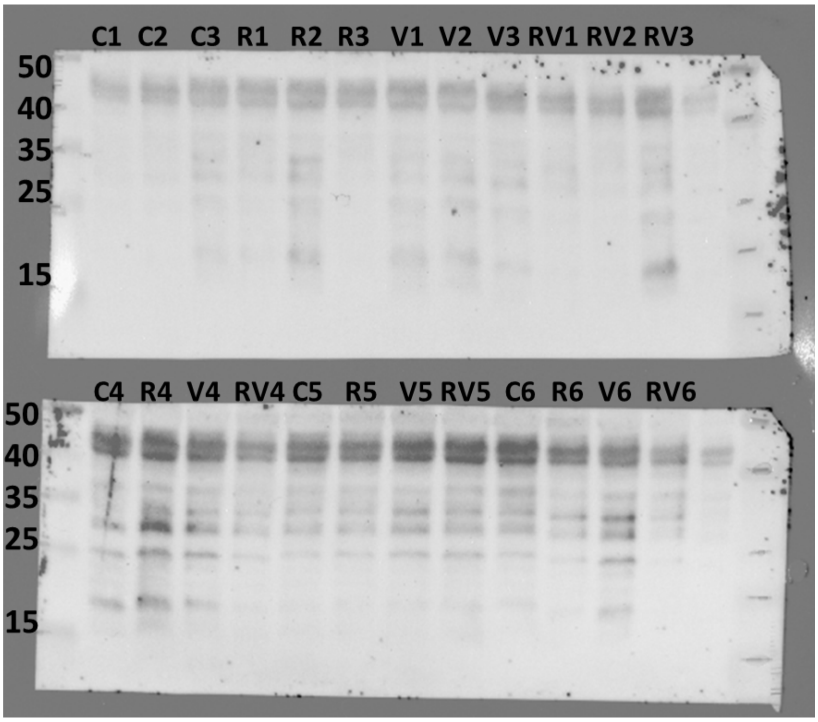

p-Creb Telencephalon (43 kDa)

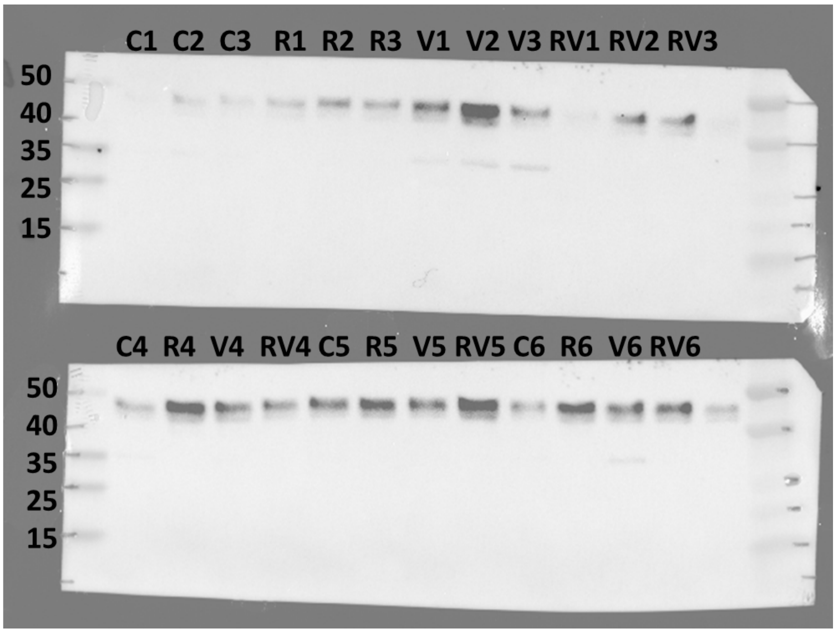

Creb Telencepahlon (43 kDa)

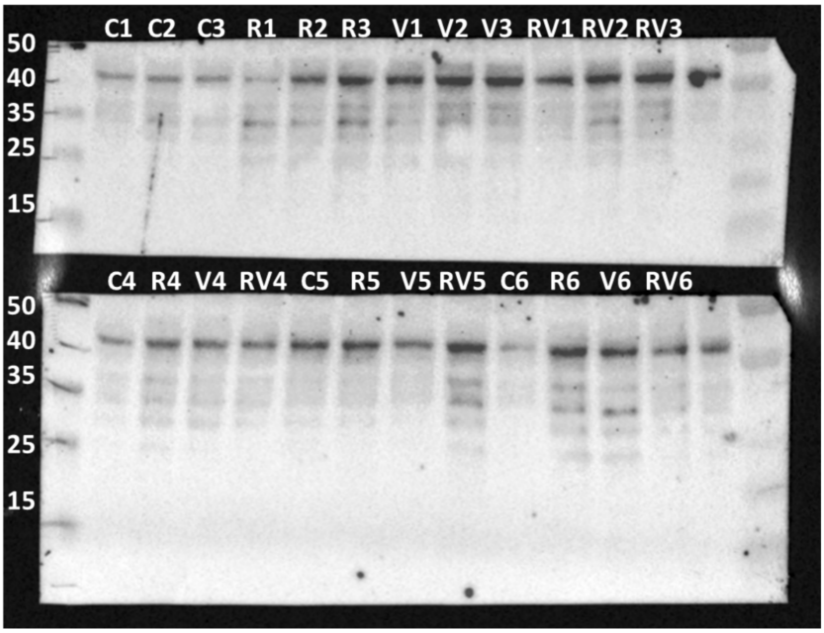

p-Foxo1 Hypothalamus (78-82 kDa)

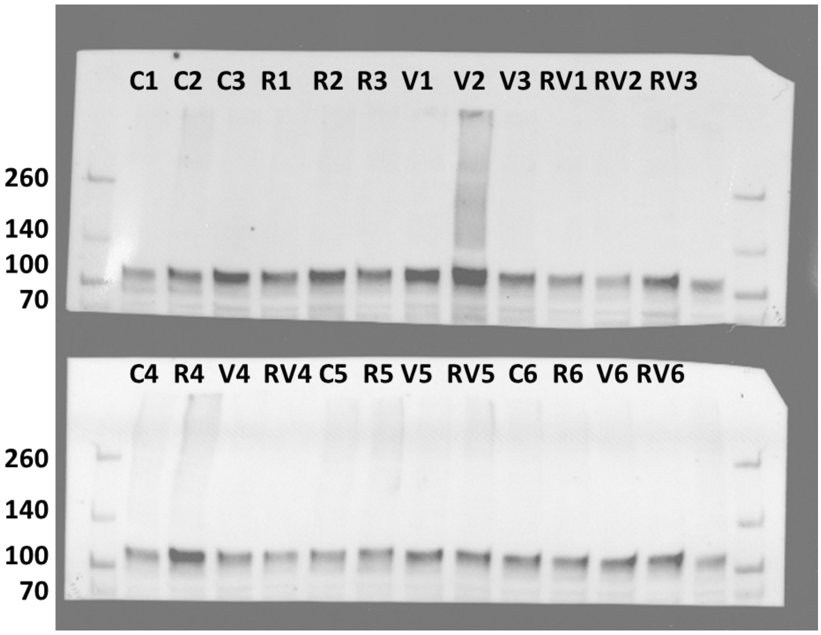

Foxo1 Hypothalamus (78-82 kDa)

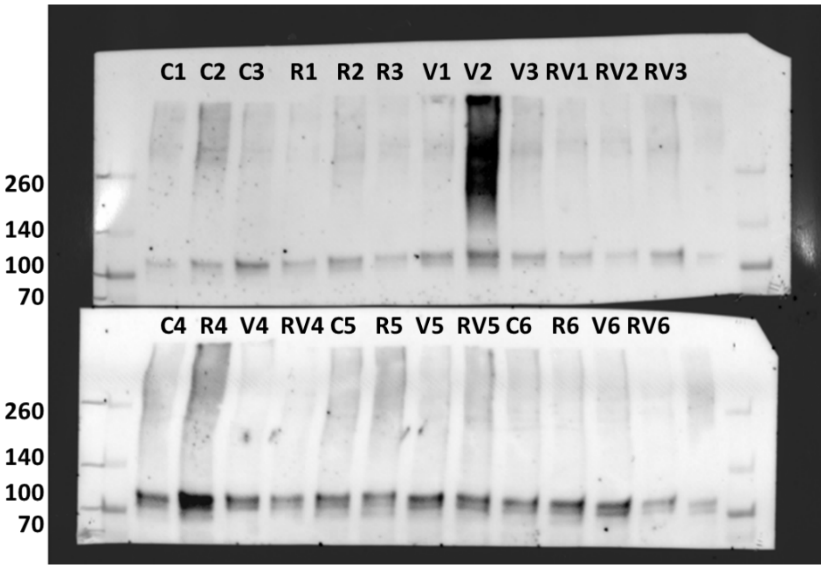

p-Foxo1 Telencephalon (78-82 kDa)

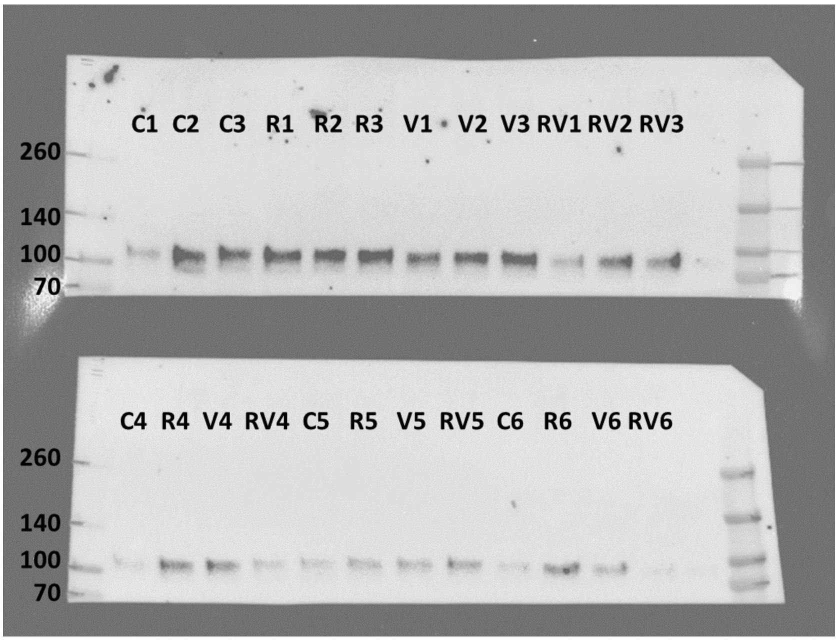

Foxo1 Telencephalon (78-82 kDa)

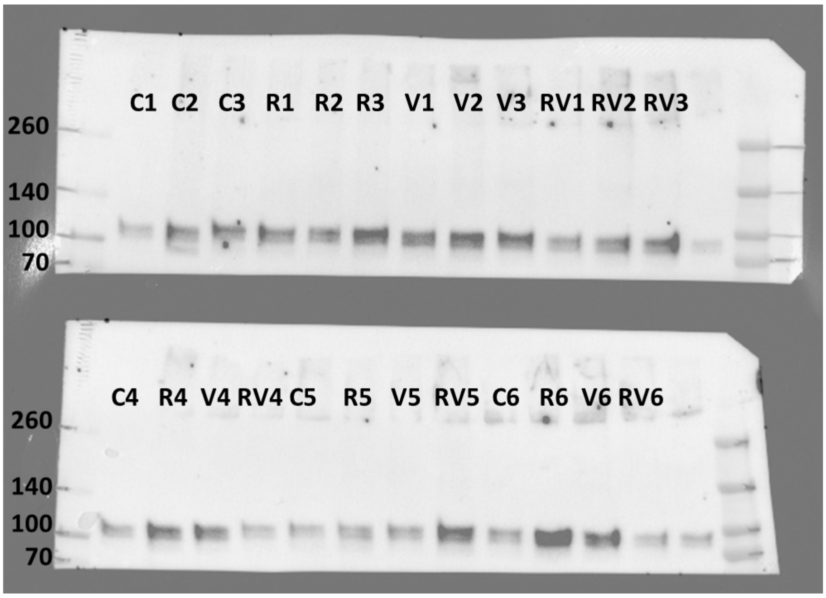

p-mTor Hypothalamus (290 kDa)

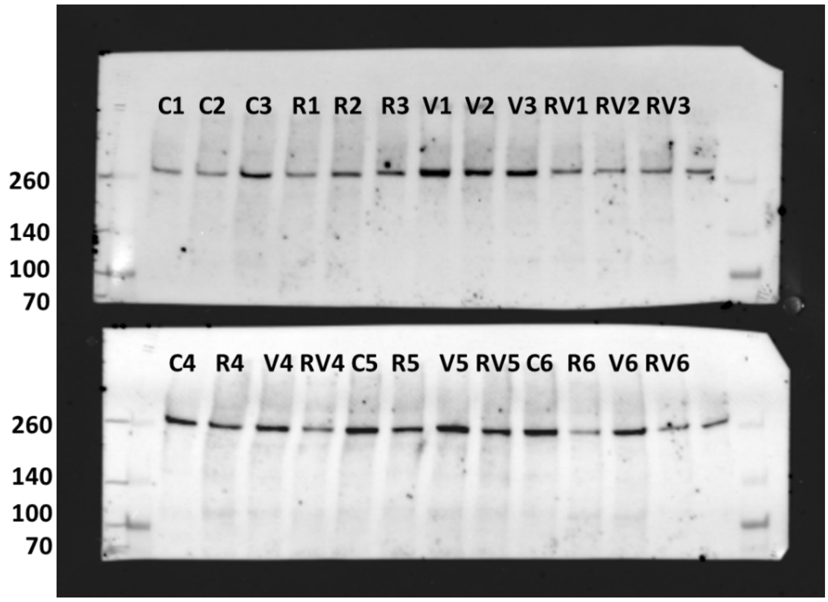

mTor Hypothalamus (290 kDa)

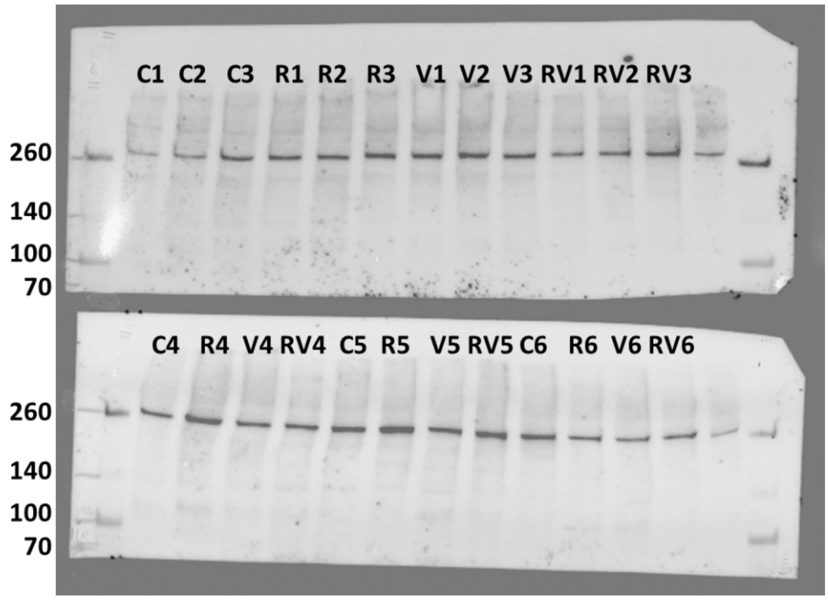

p-mTor Telencephalon (290 kDa)

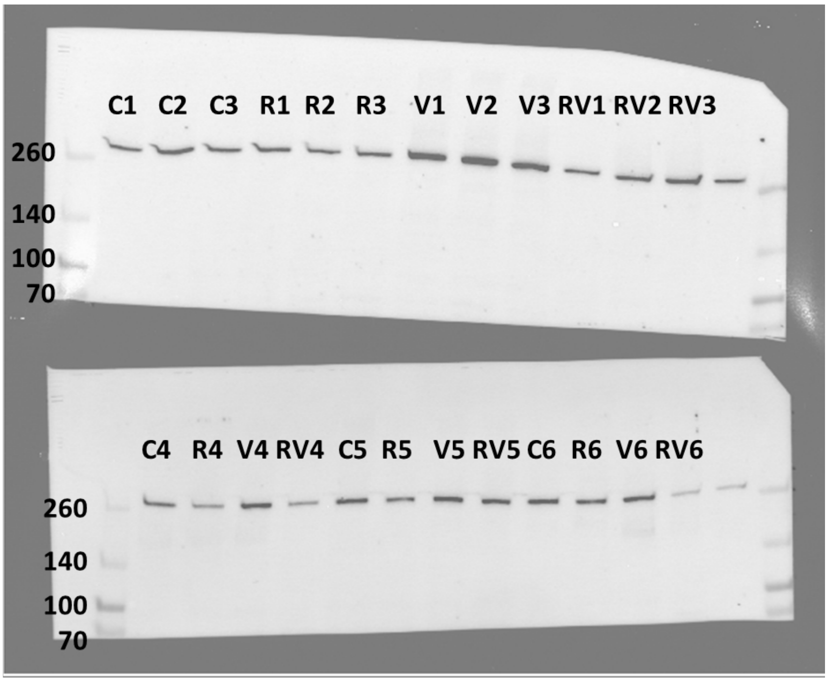

mTor Telencephalon (290 kDa)

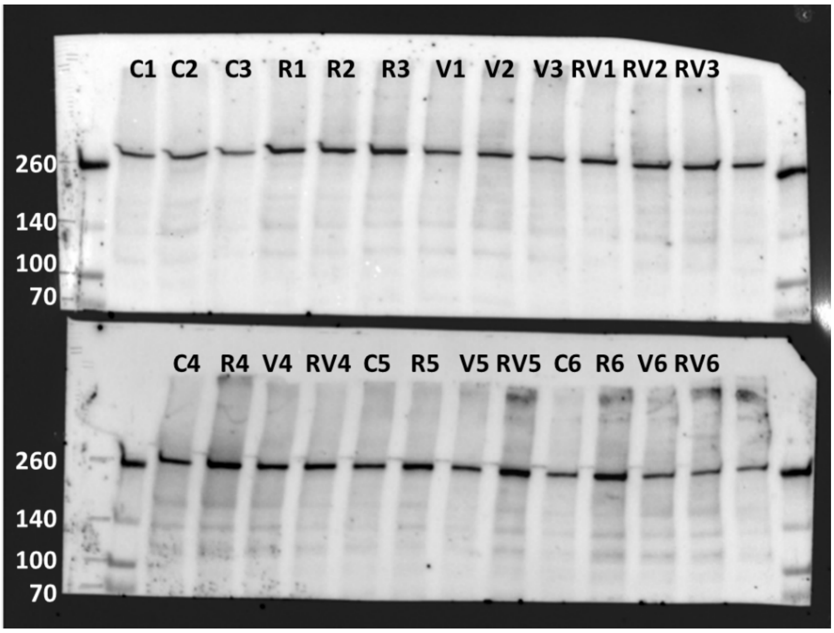

p-S6 Hypothalamus (32 kDa)

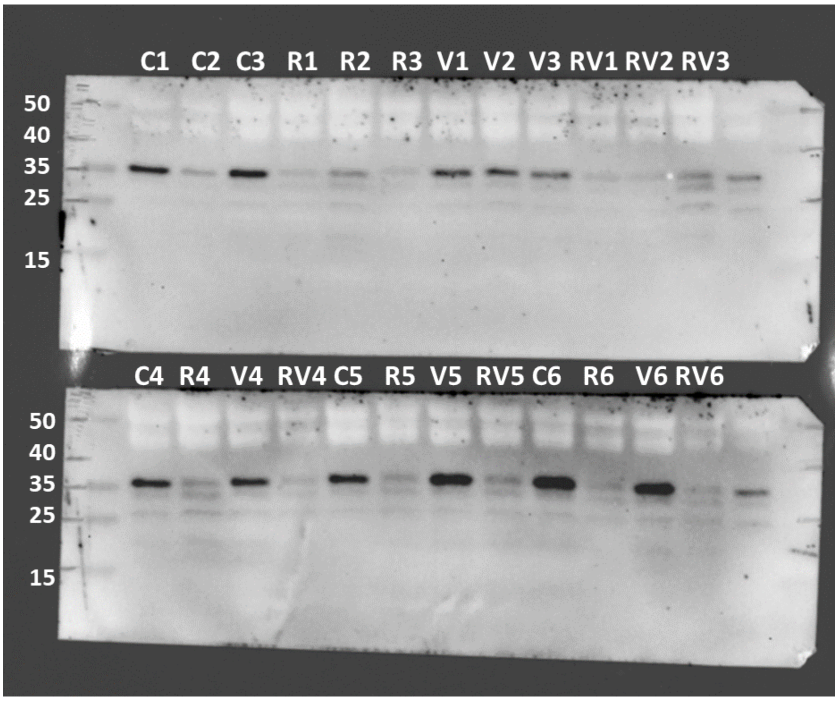

S6 Hypothalamus (32 kDa)

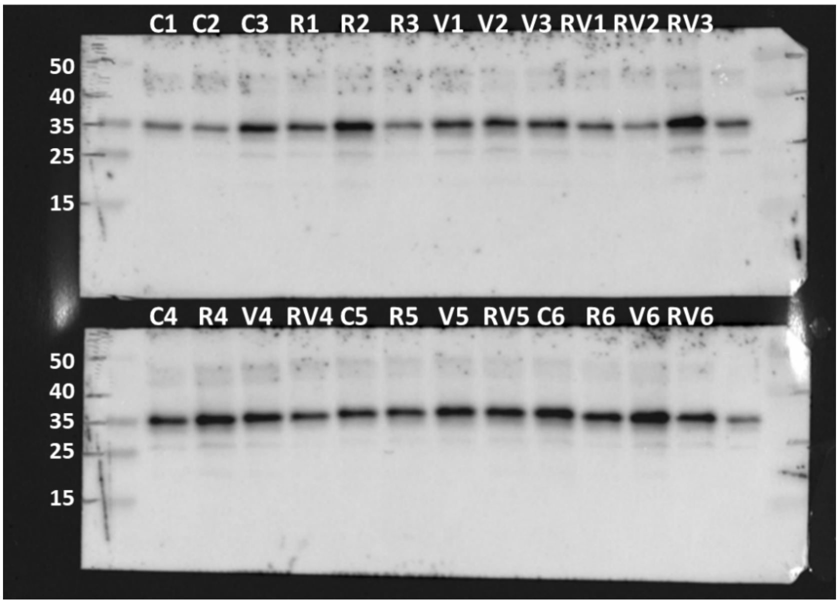

p-S6 Telencephalon (32 kDa)

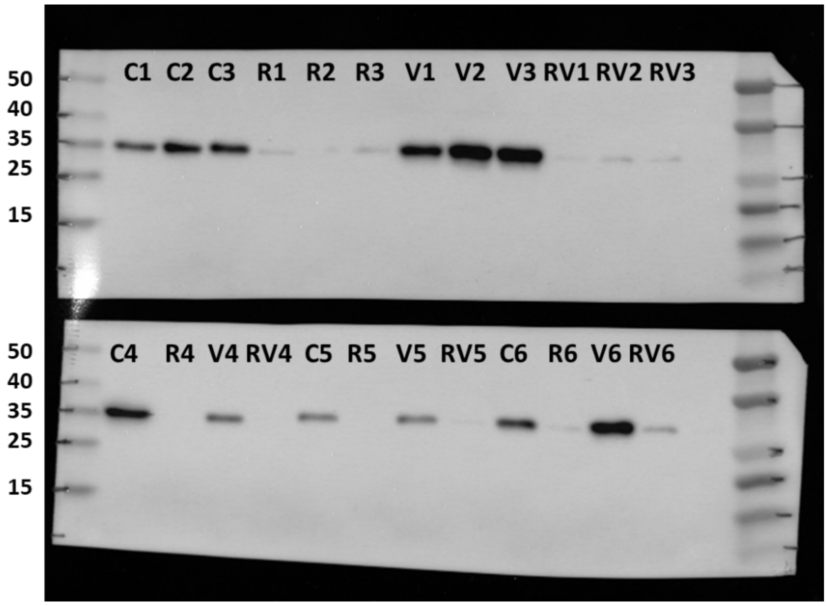

S6 Telencephalon (32 kDa)

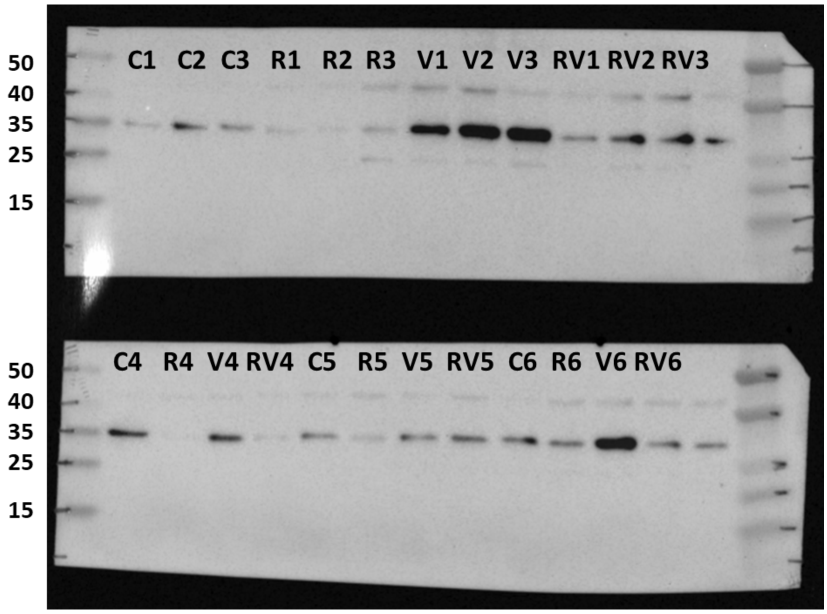

p-S6K1 Hypothalamus (70 kDa)

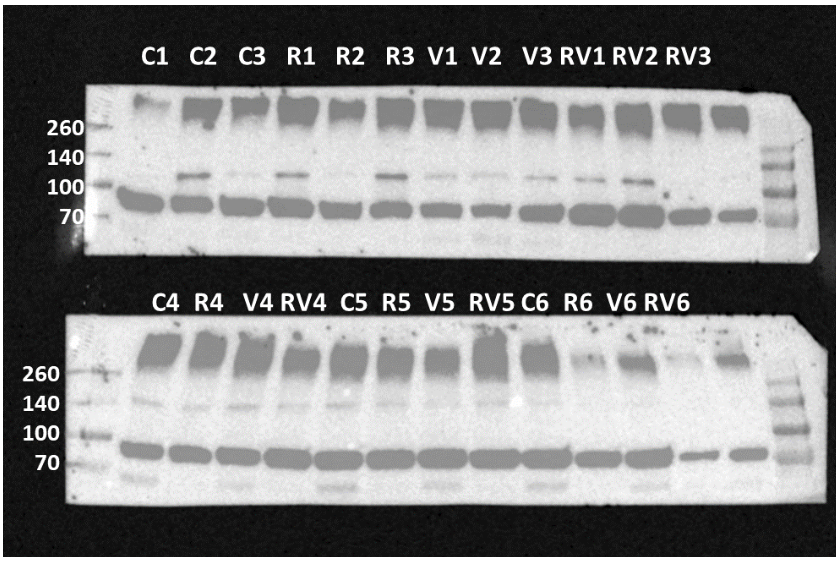

S6K1 Hypothalamus (70 kDa)

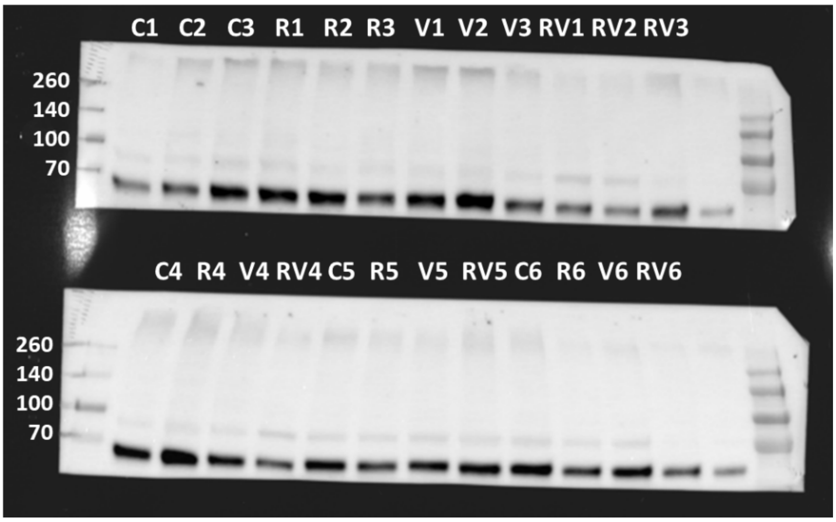

p-S6K1 Telencephalon (70 kDa)

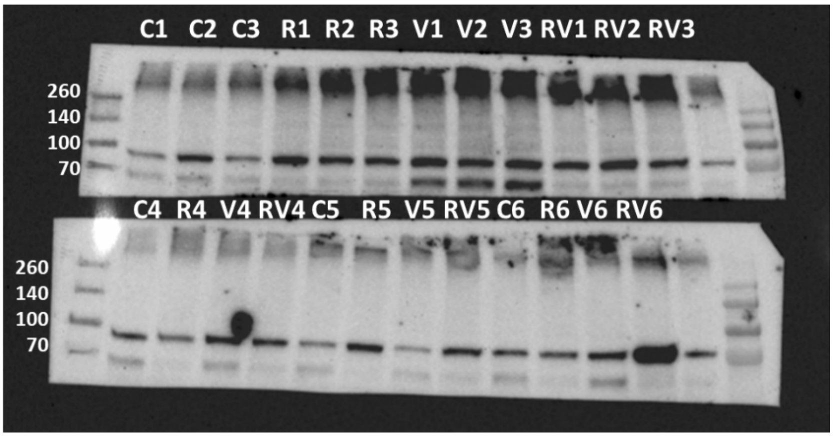

S6K1 Telencephalon (70 kDa)

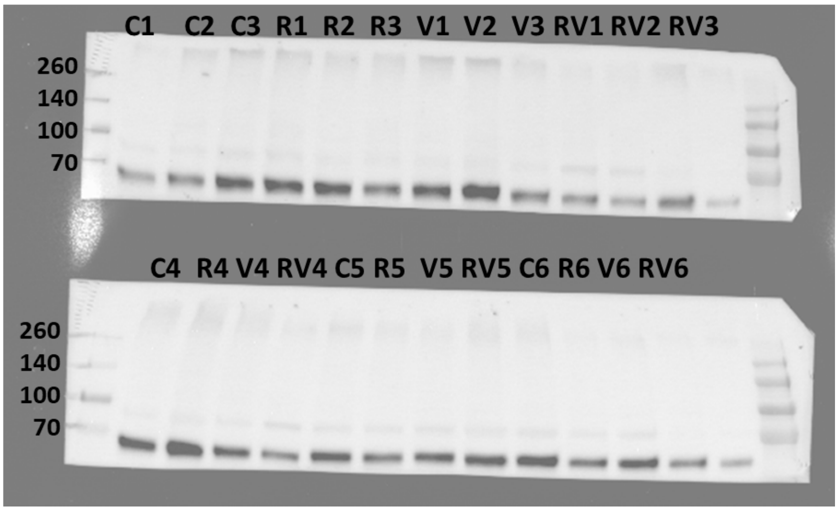

Supplement: Supplementary Materials — Original blots are included as supplementary Figure 1. [file 7509382.f1.pdf]
